# Inflammasome Molecular Insights in Autoimmune Diseases

**DOI:** 10.3390/cimb46040220

**Published:** 2024-04-18

**Authors:** Monica Neamțu, Veronica Bild, Alexandru Vasincu, Oana Dana Arcan, Delia Bulea, Daniela-Carmen Ababei, Răzvan-Nicolae Rusu, Ioana Macadan, Ana Maria Sciucă, Andrei Neamțu

**Affiliations:** 1 Department of Pharmacodynamics and Clinical Pharmacy, “Grigore T. Popa” University of Medicine and Pharmacy, 16 Universitatii Street, 700115 Iasi, Romania; monica.neamtu@umfiasi.ro (M.N.); veronica.bild@umfiasi.ro (V.B.); oana-dana.arcan@umfiasi.ro (O.D.A.); delia.bulea@umfiasi.ro (D.B.); daniela-carmen-p-ababei@umfiasi.ro (D.-C.A.); razvan-nicolae.rusu@umfiasi.ro (R.-N.R.); macadan.ioana@d.umfiasi.ro (I.M.); 2 Center of Biomedical Research of the Romanian Academy, 8 Carol I Avenue, 700506 Iasi, Romania; 3 Department of Oral Medicine, Oral Dermatology, “Grigore T. Popa” University of Medicine and Pharmacy, 16 Universitatii Street, 700115 Iasi, Romania; 4 Department of Physiology, “Grigore T. Popa” University of Medicine and Pharmacy, 16 Universitatii Street, 700115 Iasi, Romania; andrei.neamtu@umfiasi.ro

**Keywords:** inflammation, molecular mimicry, autoimmune diseases, NLRP3 inflammasome

## Abstract

Autoimmune diseases (AIDs) emerge due to an irregular immune response towards self- and non-self-antigens. Inflammation commonly accompanies these conditions, with inflammatory factors and inflammasomes playing pivotal roles in their progression. Key concepts in molecular biology, inflammation, and molecular mimicry are crucial to understanding AID development. Exposure to foreign antigens can cause inflammation, potentially leading to AIDs through molecular mimicry triggered by cross-reactive epitopes. Molecular mimicry emerges as a key mechanism by which infectious or chemical agents trigger autoimmunity. In certain susceptible individuals, autoreactive T or B cells may be activated by a foreign antigen due to resemblances between foreign and self-peptides. Chronic inflammation, typically driven by abnormal immune responses, is strongly associated with AID pathogenesis. Inflammasomes, which are vital cytosolic multiprotein complexes assembled in response to infections and stress, are crucial to activating inflammatory processes in macrophages. Chronic inflammation, characterized by prolonged tissue injury and repair cycles, can significantly damage tissues, thereby increasing the risk of AIDs. Inhibiting inflammasomes, particularly in autoinflammatory disorders, has garnered significant interest, with pharmaceutical advancements targeting cytokines and inflammasomes showing promise in AID management.

## 1. Autoimmune Diseases

### 1.1. Introduction

AIDs develop as a result of an aberrant immune response in recognizing self- and non-self-antigens. Currently, over 80 distinct autoimmune disorders have been identified [1].

Up to now, various situations have been explained where either the failure of the central tolerance selection or inadequate peripheral suppressive functions can be considered as the root cause [2].

AIDs occur when the immune system erroneously targets healthy cells and tissues within the body. This happens due to an overactive immune response. These conditions are frequently accompanied by inflammation, where inflammatory mediators like factors and inflammasomes heavily influence their progression. They affect both innate cells, such as macrophages and adaptive cells like T and B cells, leading to the initiation of autoimmune responses. Directing treatments towards inflammatory mediators and pathways presents a promising strategy for managing AIDs [3].

AIDs typically occur in three stages. At first, the immune system becomes blocked due to various factors, which causes damage to immune tolerance. During this stage, activation of the innate immune response prompts the onset of the adaptive immune response. Consequently, T and B cells begin to erroneously recognize antigens, causing disruptions in immune function. Subsequently, the second phase witnesses an excessive proliferation of innate immune cells, releasing numerous inflammatory factors. This sets off the atypical influx of T and B cells, culminating in escalating inflammation and tissue injury. The third stage revolves around regulating the formation of autoimmune responses through internal and external cellular mechanisms. This phase presents opportunities for remission and potential relapses [4,5].

### 1.2. Molecular Mimicry in Autoimmune Diseases

Exposure to a foreign antigen can cause inflammation, which in turn may lead to AIDs due to molecular mimicry from cross-reactive epitopes. These epitopes are fragments of foreign antigens that can activate CD4+ or CD8+ T cells when presented to them in the context of the major histocompatibility complex (MHC). Several mechanisms have been proposed for how infections can trigger or worsen AIDs.

A process by which the body’s immune system may unintentionally target its cells is referred to as “molecular mimicry”. This happens when foreign substances resemble the body’s cells, such as chemicals or infections. In certain individuals, this similarity can trigger an immune response, causing T or B cells to activate and ultimately leading to autoimmune reactions [6].

Damian R. introduced the expression “molecular mimicry” in 1964. He suggested that certain microorganisms might have antigenic determinants that resemble those of their host. This similarity allows the microorganism to evade the host’s immune system and avoid immune responses, effectively protecting it from the host’s defense mechanisms. In the field of immunology, the term molecular mimicry initially referred to the ability of microorganisms to mimic the structure of host antigens, which helps them evade the host’s immune system. However, the term later took on an alternative interpretation, suggesting that the antigenic components of microorganisms may elicit an autoimmune reaction that harms the host. This situation arises when there is a likeness in structure between self-structures and a pathogen or metabolite, which can arise from common amino acid sequences (linear or mimotope) or comparable conformational structures shared between the pathogen and the self-antigen [7].

In the early 1980s, researchers proposed the concept of molecular mimicry as a potential cause of autoimmune diseases (AIDs). This phenomenon happens when peptides from viral or bacterial proteins bear a resemblance to self-peptides in terms of sequence or structure. Such a similarity has the potential to trigger immune responses that attack the body’s tissues, leading to the production of self-reactive antibodies [8,9].

In infection-induced autoimmunity, postulated mechanisms include the activation of autoreactive T cells through encounters with pathogens sharing epitopes or cross-reacting with self-antigens, a phenomenon known as molecular mimicry [10]. Additionally, autoreactive T cells can be activated de novo by the release of sequestered antigens during tissue damage caused by virus-specific T cells, termed epitope spreading [11]. Furthermore, autoreactive T cells with specific Vβ receptors can be stimulated by virus-encoded superantigens [12].

In some cases, the organism may launch an autoimmune response after infection. This happens when the immune system, while fighting the pathogen, also mistakenly attacks the body’s cells. This is known as molecular mimicry, which means that the same antigenic determinants found in the infecting pathogen are similar to those present in the host’s cells.

Various mechanisms have been suggested to elucidate how pathogens can stimulate and amplify autoreactive T cells, resulting in AIDs [13]. Molecular mimicry is a mechanism where a microbial antigen may contain an epitope that resembles the structure of an autoantigen epitope [14,15].

**Figure 1:**
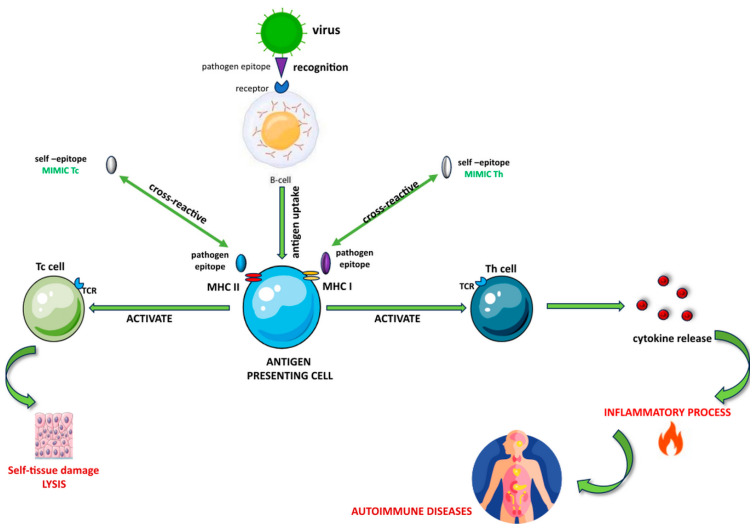
Infection-induced autoimmunity by molecular mimicry. Legend: MHC, Major histocompatibility complex; TCR, T cell receptor.

To initiate a response, T cells require a signal from an antigen-presenting cell (APC). This signal is generated by the recognition of an antigenic peptide bound to an MHC molecule on the surface of the APC. This recognition is crucial for the activation of T cells, which then proceed to carry out their function in the immune response (Figure 1). The T cell antigen receptor can recognize the antigen−antibody complex in a flexible manner, allowing it to identify a wide range of foreign antigens. However, this flexibility can also create a risk of T cell antigen receptors mistakenly cross-reacting with self-antigens, which can trigger autoimmunity. Immunological tolerance can protect against autoimmunity. It is a state where the individual is unable to develop an immunologic response to a self-antigen [16]. The immune system uses different mechanisms, such as the MHC, to recognize and combat foreign pathogens [17].

Inflammatory responses in tissues triggered by pathogens can lead to the local activation of APCs, thereby improving the processing and presentation of self-antigens. This, in turn, initiates T cell priming, followed by the activation and expansion of additional specificities (known as epitope spreading) [18]. By binding to MHC class II molecules, viral and bacterial superantigens can activate resting autoreactive T cells irrespective of their specificity [12].

These T cells exhibit reactivity towards both foreign and self-antigens, making the host vulnerable to external factors that can trigger an autoimmune reaction [17]. Recent studies have delved into the B cell response associated with the production of autoantibodies. Autoimmune recognition of self-antigens is indicated by the presence of autoantibodies [6]. The B cells contribute to multiple forms of autoimmune inflammation [19] and can utilize multiple mechanisms in autoimmune responses, including the production of antibodies and cytokines [20].

Chronic AIDs occur when the immune system attacks the body’s own tissues, mistaking them for foreign substances. This process can cause inflammation and damage to specific organs and tissues [21]. Although the genetic background of the host contributes to the immune response towards the self, there is evidence indicating that infectious agents, both viral and bacterial, are the primary environmental causes of AIDs [17].

#### 1.2.1. Molecular Mimicry in Multiple Sclerosis

Multiple sclerosis (MS) stands as the prevalent inflammatory AID affecting the central nervous system (CNS). The gradual disability stemming from brain lesions occurs due to the immune-mediated deterioration of the protective myelin sheath surrounding neurons [22]. The exact trigger of MS is not known, but studies have shown that viral infections may play a significant role, with one possible explanation for its development being infections engaging in molecular mimicry [11].

The cause of this condition remains unidentified; however, a combination of genetic and environmental factors plays a role in increasing the likelihood of developing MS [23,24]. Although many viruses have been linked to MS, no single virus has been proven to be the direct cause of this condition. Moreover, it is believed that MS has an autoimmune component. Molecular mimicry is a proposed explanation for the diverse pathology and origins of MS. It occurs when pathogenic peptides exhibit sequence or structural resemblances to self-antigens. [25]. A virus can harm the immune system directly or make it more active, which could result in an altered or intensified immune response that may attack the body’s proteins. It is unclear whether a virus could trigger immune system issues that lead to demyelination, whether it could become latent in the CNS and reemerge during times of stress, and whether it could cause damage at this level [26].

#### 1.2.2. Molecular Mimicry in Autoimmune Hepatitis

Autoimmune hepatitis (AIH) is a serious liver condition initiated by the immune system targeting and attacking the liver [27].

One of the main criteria used to diagnose AIH and its subtypes is the detection of specific autoantibodies working against liver autoantigens [28,29].

In some cases, when pathogens share a similar structure with the host, the immune response can be triggered and affect both the pathogen and the host. This is known as molecular mimicry and can lead to the breakdown of self-tolerance and the appearance of AIDs. There have been reports of sequence homologies between autoantigens and pathogens associated with AIDs, as well as the presence of cross-reactive antibodies and/or T cells [17,30].

The development of this illness encompasses T-cell-induced harm, an imbalance between regulatory and effector cells, and the breakdown of immune tolerance [31,32].

#### 1.2.3. Molecular Mimicry in Autoimmune Thyroid Disease

Autoimmune thyroid disease (AITD) occurs when the immune system mistakenly attacks the thyroid gland, causing damage to the thyroid cells and affecting its ability to produce hormones. One cause of this disease is molecular mimicry, which happens when certain molecules in the thyroid cells resemble molecules found in bacteria or viruses. This similarity may confuse the immune system and trigger it to attack both the foreign invaders and the thyroid cells, leading to AITD.

Several infectious agents have been linked to the onset of AITD, e.g., *Yersinia* spp., *Helicobacter* spp., *Bartonella henselae*, the influenza virus, herpesvirus, retrovirus, the hepatitis C virus (HCV), and staphylococcal infection [33,34].

#### 1.2.4. Molecular Mimicry in Systemic Lupus Erythematosus

Systemic lupus erythematosus (SLE) is a complex AID that can affect different organs and systems in the body. This condition arises from a blend of genetic, epigenetic, and environmental elements that provoke an atypical immune reaction towards the body’s tissues, leading to chronic inflammation [35].

It has been found that infectious agents can play a significant role in triggering and aggravating autoimmunity in individuals with SLE. Several studies have investigated the possible link between infectious agents and the development of SLE, and some viruses, such as the *Epstein–Barr* virus (EBV), parvovirus B19, human T-lymphotropic virus 1, cytomegalovirus, and HCV, have been identified as potential contributors [36,37].

#### 1.2.5. Molecular Mimicry in Rheumatoid Arthritis

Molecular mimicry is thought to play a role in the development of rheumatoid arthritis (RA). In susceptible individuals, the similarity between the amino acid sequences of certain harmful microorganisms and human proteins may trigger an immune response that leads to AIDs like RA. Additionally, some pathogens may cause RA by producing pro-inflammatory cytokines that activate T cells and promote the production of autoantibodies. However, the exact role of molecular mimicry in the pathogenesis of RA is still unclear, and further investigation is necessary to understand the mechanisms involved. Several environmental factors, including infectious diseases, are associated with the development of RA in genetically predisposed individuals [38].

Both clinical and experimental research suggest that microorganisms like *Escherichia coli*, *Porphyromonas gingivalis*, *Proteus mirabilis*, and EBV contribute to the progression of RA [39,40]. Additionally, various infectious agents contain peptides resembling the human proteome, with Mycobacterial heat shock proteins showing similarities to human heat shock proteins [6].

Molecular mimicry plays a significant role in autoimmune diseases by triggering immune responses against self-components due to antigen sharing between pathogens and hosts. This phenomenon can lead to autoimmunity, as seen in AIH, AITD, SLE or RA.

## 2. Autoimmune Disorders Pathogenesis

The term AIDs refers to a diverse group of conditions in which the immune system mistakenly targets and attacks the body’s own healthy cells and tissues. Despite sharing certain common mechanisms, AIDs can manifest in a wide range of clinical symptoms that can vary from life-threatening organ failure to minor laboratory abnormalities that are easy to miss [5].

**Figure 2:**
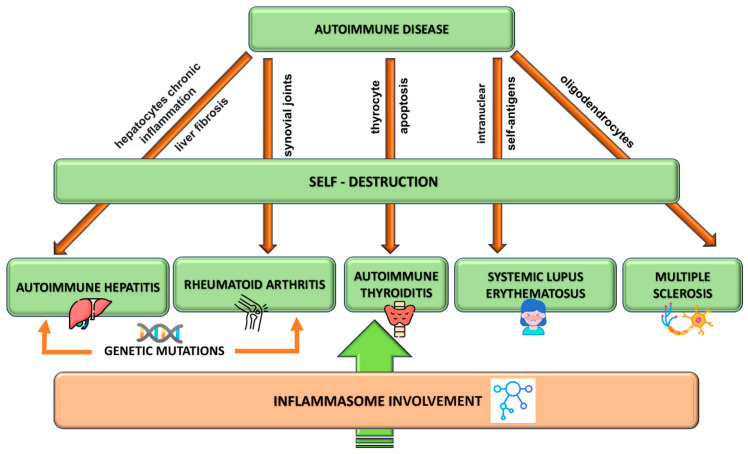
Involvement of inflammation in autoimmune diseases.

Figure 2 illustrates how abnormal inflammation is a major factor in both acute and chronic conditions. Additionally, there is compelling evidence linking chronic inflammation to the pathogenesis of AIDs.

### 2.1. Multiple Sclerosis Pathogenesis

MS affects the CNS and can cause significant physical or cognitive impairments, as well as neurological issues, particularly affecting young adults [41]. The degeneration of the nerve fiber protection, leading to the formation of CNS plaques, primarily stems from multiple areas of inflammation, caused by the infiltration of T cells and macrophages, alongside the demise of oligodendrocytes. These plaques contain inflammatory cells and byproducts, as well as demyelinated and severed axons. Furthermore, as a consequence of this process, astrogliosis occurs in both white and gray matter [42].

In MS, damage arises from the inflammation of white and gray matter tissues within the CNS due to the infiltration of immune cells and cytokines. Various research studies highlight the significance of T helper (Th) cells (also referred to as CD4+ T cells) and adaptive immune responses, which are initiated through the interaction between APCs and T lymphocytes, in the initiation and progression of MS [43,44].

When a pathogen invades the body, it triggers a response from the immune system. This involves the binding of pathogen-associated molecules to Toll-like receptors on APCs. As a result, specific cytokines, such as interleukin (IL)-12, IL-23, and IL-4, are produced. These cytokines play a crucial role in the differentiation of CD4+ T cells into different phenotypes, namely Th1, Th2, or Th17. Each of these phenotypes can release specific cytokines. For example, Th1 cells produce proinflammatory cytokines such as interferon-gamma (IFN-γ) and tumor necrosis factor-alpha (TNF-α) [45]. Th17 cells are a subset of CD4+ T cells that produce multiple cytokines, such as IL-17, IL-21, IL-22, and IL-26. These cytokines are capable of inducing inflammation [46]. While the exact origin of MS remains unclear, genetic tendencies, in conjunction with environmental factors, significantly impact the development of this condition.

### 2.2. Autoimmune Hepatitis Pathogenesis

AIH is a condition involving liver inflammation that triggers necrosis-resembling debris in the liver portal area, marked by high levels of gamma globulins and numerous autoantibodies [47]. The development of AIH is intricately linked to genetic predispositions, immune irregularities, and various external elements like viral infections, environmental triggers, and medications that can induce immune system dysfunction [48].

AIH is caused by the immune system attacking liver cells due to a lack of immune tolerance to hepatocyte antigens. This results in the damage of liver tissue cells facilitated by self-targeting T cells [49]. It is believed that a specific part of a liver protein (known as an epitope) attaches to a particular part of a type of immune system protein, known as a HLA class II antigen (known as a paratope), and is then displayed on the surface of certain cells in the immune system. It is possible that a viral infection or exposure to certain toxins could result in changes to these liver protein epitopes, which may then trigger an immune response, potentially due to molecular mimicry [50].

While the precise triggers for AIH remain unclear, it is believed to commence with an immune response directed against the body’s own liver antigens [51].

There are two types of cytotoxic T cells (CTLs) that can cause liver injury. These are classified as CD4 CTL and CD8 CTL. CTLs bring about target cell death through two primary molecular mechanisms. The perforation-based degranulation pathway is mainly associated with CD8 CTL, which leads to quick target cell death. On the other hand, the Fas-mediated hepatocellular apoptosis involves the Fas ligand (FasL) on the CTL binding with the target cell-associated Fas antigen, causing apoptosis of target cells. This mechanism primarily affects CD4 CTL [47]. Fas-mediated hepatocellular apoptosis could be involved in hepatocyte injuries during the development of AIH [52].

### 2.3. Autoimmune Thyroid Disease

AITDs are prototypical organ-specific AIDs, but the mechanisms triggering the autoimmune response to the thyroid are still unclear [53]. Graves’ disease (GD) and Hashimoto’s thyroiditis (HT) are the predominant culprits behind thyroid gland malfunctions. These disorders stem from intricate interplays of genetic and environmental elements. They involve the immune system targeting thyroid antigens because of self-reactive lymphocytes that bypass self-tolerance mechanisms [54]. This leads to a disruption in self-tolerance towards thyroid antigens, resulting in the production of antibodies and the infiltration of lymphocytes [55].

Cell-mediated autoimmune responses predominantly drive HT, while GD is a result of humoral autoimmunity [56]; however, in AITD, both cellular and humoral immune responses are interconnected and function jointly [57]. Traditionally, HT has been viewed as a Th1-driven condition, but this viewpoint has evolved with the identification of additional Th cell subsets, such as Th17 cells [58]. Traditionally, HT has been categorized as a Th1-mediated disease, but new research has described the involvement of other Th cell subsets, including Th17 cells, in different types of AIDs, which were considered until now to be Th1-dependent diseases [59]. This uncontrolled Th17 cell response leads to the thyroidal infiltration of inflammatory B and T cells, which can cause goiters initially [60].

Researchers believe that AITDs are complex and multifactorial diseases caused by a combination of genetic, hormonal, and environmental factors that trigger inappropriate immune responses against the thyroid gland, leading to the onset of a persistent autoimmune response [54].

### 2.4. Systemic Lupus Erythematosus

SLE arises from intricate dynamics between environmental factors and genetic predispositions that lead to epigenetic alterations, impacting gene expression linked to the disease’s emergence. This results in the immune system encountering self-antigens due to cell damage from infections and environmental factors, leading to the activation of T and B cells [61]. Exposures to ultraviolet radiation type B (UVB) or infections can influence the immune systems of people with genetic predispositions, initiating an inappropriate immune response [62]. The release of cytokines, the complement system activation, and production of autoantibodies subsequently lead to damage in various organs [63].

Both the innate and adaptive immune systems play a role in the development of SLE.

In the adaptive immune system, T and B cells are critical players. Antigens from apoptotic and damaged cells are presented to T cells, which exhibit altered gene expression and cytokine production in SLE. T cells produce less IL-2, affecting regulatory T cell function, while elevated levels of IL-6, IL-10, IL-12, IL-17, IL-21, and IFN-γ influence mononuclear and T cell production, leading to impaired T cell function [64]. Th17 cells, as IL-17 producers, contribute to inflammation and tissue damage by driving neutrophil activity and B cell function [65]. The activated autoreactive B cells lead to the production of autoantibodies, a defining characteristic of SLE. The activation of B cells by Toll-like receptors (TLRs) interacting with deoxyribonucleic acid (DNA) and ribonucleic acid (RNA) involves nucleic acid and protein complexes as key antigens. The negative impact of autoantibodies is explained by depositing immune complexes, activating the complement system, and disrupting cell functions. This process promotes apoptosis and the production of additional cytokines [66].

B cells exacerbate SLE pathogenesis through antigen response and autoantibody production. Pathways involved in B cell dysregulation include TLR stimulation, particularly TLR7 and TLR9, and activation via other factors, leading to tolerance breakdown. Abnormal B cell activation is further influenced by polymorphisms in genes affecting receptor signaling. An imbalance between pro-survival and pro-apoptotic signals in these cells is also implicated in the disease’s development [67,68].

Complement dysfunction is believed to exacerbate the progression of SLE by hindering the removal of apoptotic cells and immune complexes, heightening the activity of autoreactive CD8+ T cells and triggering organ inflammation where immune complexes are deposited [69]. A stark absence of C1q leads to a failure in clearing apoptotic cells, resulting in autoantibodies and a lupus-like condition, highlighting the complement system’s critical role in preventing SLE [70].

### 2.5. Rheumatoid Arthritis Pathogenesis

RA is a systemic AID that primarily impacts joints. Macrophages, T and B cells, fibroblasts, chondrocytes, and dendritic cells are involved in the etiology and pathogenesis of RA [71].

The disease is marked by inflammation of the synovium, abnormal cell growth, the generation of autoantibodies (including anti-citrullinated protein and rheumatoid factor), and the degradation of bone and cartilage, which could lead to deformities in the skeletal structure [72].

Genetic and environmental influences are considered key factors in RA development. T cells, B cells, and interactions with pro-inflammatory cytokines are essential in the pathophysiology of RA [73]. Immune modulators, such as cytokines and effector cells, as well as signaling pathways are involved. The cytokines TNF-α and IL-6 are directly involved in this mechanism, although IL-1 and IL-17 might also hold significance in the progression of the disease [74].

The onset typically involves activation of the innate immune response, wherein dendritic cells respond to external and self-antigens. APCs present arthritis-associated antigens to T cells, while CD4+ T cells secreting IL-2 and IFN-γ infiltrate the synovial membrane [75].

Pro-inflammatory cytokines, such as IL-6 and TNF-α, are pivotal in RA’s progression [76,77]. TNF-α activates various cells, including chemokines, leading to endothelial cell adhesion molecule expression, neoangiogenesis, T cell suppression, and pain induction [78]. Conversely, IL-6 stimulates leukocytes or autoantibody production. IL-1, similarly important in RA, activates osteoclasts and chondrocytes. Additionally, RA progression involves intracellular signaling molecules, such as Janus kinase, in the synovium regulating inflammatory agents like IFNs and cytokines [79].

Despite extensive research, the pathophysiology of RA remains incomplete, and there is no permanent cure for the disease.

AIDs result from genetic, epigenetic, and environmental factors causing immune system imbalances, leading to inflammatory reactions against self-antigens, either systemically or in specific organs.

## 3. Inflammasome Structure and Functioning

The components of an inflammatory complex, known as an apoptosis-associated speck-like protein containing a caspase-recruitment domain (ASC) and proinflammatory caspase-1, are three proteins known as inflammasomes that are found inside cells and function as molecular factories [80].

They are vital in activating inflammatory caspases and releasing pro-inflammatory mediators, being assembled in response to infection and cellular stress [81]. Many studies, both experimental and theoretical, have been concerned with the three-dimensional structure of inflammasomes on an atomic scale, as well as its modulation mechanisms by different natural compounds, like chondroitin sulfate, or synthetic therapeutic peptides and hydrogels [82,83,84,85,86,87].

The inflammasome includes a pattern recognition receptor (PRR) responsible for sensing pathogen-associated molecular patterns (PAMPs) or damage-associated molecular patterns (DAMPs), procaspase-1, and an adaptor molecule like ASC, which connects the sensor with procaspase-1 [88].

Inflammasomes can be classified into nucleotide-binding domain (NBD)-like receptors (NLR), absent in melanoma 2-like (AIM2) receptors (ALR), and pyrin inflammasomes. Within these classifications, they are categorized as canonical types, including the nucleotide-binding domain, leucine-rich-containing family, pyrin domain-containing (NLRP)-1, NLRP-3, NLR family CARD domain-containing protein 4 (NLRC4), AIM2, and non-canonical forms like Caspase-4/5/11.

### 3.1. Canonical Inflammasomes

Different stimuli are known to activate different types of NLRs, such as NLRP1, NLRP3, NLRC4, and the non-NLR family pathogen receptor AIM2 [89,90]. Activating an inflammatory caspase, caspase-1, these so-called “canonical inflammasomes” cause pro-inflammatory cytokines, IL-1β and IL-18, to mature and secrete, and they also trigger pyroptosis, an inflammatory type of pathogen-induced programmed cell death [80,91].

NLRP1, NLRP3, and NLRC4 are components of inflammatory cytoplasmic dimers. They possess a central NBD, a C-terminal leucine-rich repeat (LRR), and a pyrin or caspase activation and recruitment domain (CARD). These receptors cause pyroptosis, an inflammatory type of programmed cell death that happens during pathogen infection, when they are activated. Pro-inflammatory cytokines like IL-1β and IL-18 are released as a result.

The receptors (PRRs) can sense different stimuli, with AIM2 recognizing intracytoplasmic DNA and NLRP3 responding to PAMPs, DAMPs, and even environmental chemicals like silica [88].

### 3.2. Non-Canonical Inflammasomes

When intracellular lipopolysaccharide (LPS) from Gram-negative bacteria is recognized by caspase-11, it can trigger pyroptosis and the release of IL-1β and IL-18 from macrophages, which in turn can trigger inflammatory responses [92]. While the inflammatory response triggered by caspase-11 is comparable to that triggered by NLRs and AIM2-inflammasomes, it differs from these classical inflammasomes in terms of molecular mechanism and composition during macrophage-mediated inflammatory responses. Consequently, this scaffold for caspase-11 is regarded as a “non-canonical inflammasome” [91,93].

The lipid A moiety of intracellular LPS is directly bound by the CARD motif of caspase-4, -5, and -11, and IL-1 and IL18 are secreted as a result of the caspases being activated by this binding [88,94].

Activation of the inflammasome plays a critical role in the body’s defense against pathogens. Nonetheless, recent studies have revealed that this mechanism is also involved in the development of several diseases that have an inflammatory component [95].

When macrophages detect external PAMPs, which are components of invading pathogens, they trigger an inflammatory response through their molecular receptors (known as PRRs) on the cell surfaces [96].

When exposed to different extracellular and intracellular PAMPs and stimuli, PRRs cause inflammatory reactions. Numerous intracellular PRR subtypes have been identified, such as caspase-11, retinoic acid-inducible gene I (RIG-I)-like receptors (RLRs), NLRs, LRRs, and AIM2 [97]. The NLR family of proteins, which is a group of intracellular PRRs belonging to the innate immune system, is crucial for recognizing different kinds of pathogen- and danger-associated molecular patterns (DAMPs and PAMPs, respectively) [98].

The NLR proteins, NLRP3 (also called NALP3 or cryopyrin), NLRP1, NLRC4, and the IFI200 family member AIM2, are activated to form inflammasomes. In reaction to particular stimuli, the appropriate NLR or AIM2 can oligomerize to form a scaffold that activates caspase-1. After that, active caspase-1 breaks down the proinflammatory IL-1 family of cytokines into their bioactive forms, IL-1β and IL-18, which cause pyroptosis, an inflammatory cell death mechanism [99,100].

Although there are few genetic connections between AIDs and the inflammasome-inducing NLRs, this does not rule out the possibility that inflammasome activation plays a part in the development of these illnesses [95].

Numerous AIDs and autoinflammatory conditions, including neurodegenerative illnesses, have been connected to inflammasomes. Inflammasomes can be causal or contributing factors in the onset of inflammatory disease, and they can also exacerbate the pathology in response to host-derived factors [101].

Inflammasome products, such as IL-1b and IL-18, can influence adaptive immunity, suggesting a role for the inflammasome in some AIDSs. This is in line with the range of endogenous danger signals that trigger NLRs [80]. The activation of an NLR is believed to be connected to the adaptive immune responses through the amplification of T and B cell responses by IL-1b and IL-18, which could be considered as a vital link in this process [95].

### 3.3. Inflammasome Activation

A range of stimuli linked to infection or cellular stress can cause an inflammatory caspase-1 assembly, which in turn activates the protein [100,102]. After detecting PAMPs or DAMPs, inflammatory macrophages gather in the cytosol [90].

Inflammasomes play a crucial role in triggering inflammatory responses in macrophages. However, chronic inflammatory responses can increase the risk of developing an inflammatory condition or AIDs. This suggests that uncontrolled and repeated activation of inflammasomes may be a significant factor in the development of these conditions [97]. When inflammasomes are active, they can start a chain reaction of inflammation in the body. This can cause the immune system to create new types of T cells and produce different cytokines that may lead to AIDs.

Out of the different NLRP varieties, NLRP3 is the most researched, and it plays a vital role as a PRR in both innate immunity and inflammation. It detects a variety of harmful stimuli, including pathogens like bacteria and viruses, as well as signals of tissue damage. Once these signals are detected, the NLRP3 inflammasome is activated, leading to the activation of caspase-1, which results in pyroptosis and the production of important proinflammatory cytokines, such as IL-1β and IL-18. This series of events contributes to the initiation of inflammatory responses [103].

Distinct inflammasomes exhibit varied activation mechanisms. For example, AIM2 can only be activated by DNA from viruses or bacteria, whereas NLRP3 can react to a wide range of agonists that are widely available and have distinct structural and chemical characteristics. This makes NLRP3 the most versatile inflammasome with the widest functional range in both the innate and adaptive immune systems [99,104].

### 3.4. NLRP3 Canonical Activation

Although there are fundamental differences amongst inflammasomes depending on stimuli, canonical inflammasomes typically function as a scaffold to recruit the inactive zymogen pro-caspase-1 [101]. After pro-caspase-1 is assembled into active caspase-1, mature and active IL-1β and IL-18 are produced from pro-IL-1β and pro-IL-18, respectively. A form of inflammatory cell death called pyroptosis can result from activation of an inflammatory caspase-1, and active caspase-1 allows for the non-conventional secretion of many cytosolic proteins [94].

Among the inflammasomes, the NLRP3 inflammasome has the best characterization. While the amounts of ASC and pro-caspase-1 in cells do not change, the quantity of NLRP3 in resting myeloid cells (e.g., human monocytes) is insufficient in regard to allowing for activation in response to stimuli, suggesting that NLRP3 is a factor regulating the NLRP3 inflammasome’s activation [105].

The NLRP3 inflammasome can be activated through a variety of mechanisms, including adenosine triphosphate (ATP), reactive oxygen species (ROS), K^+^ efflux, and lysosomal disruption. By opening the P2X purinoceptor 7 (P2X7R) channel, high extracellular ATP levels also cause a K^+^ efflux. Preincubation with certain antioxidants blocks the NLRP3 inflammasome activation, which is triggered by numerous NLRP3 triggers that increase mitochondrial ROS production [94]. Never in mitosis gene A (NIMA)-related protein kinase 7 (NEK7), through direct interaction with NLRP3, is required for the formation of the NLRP3 inflammasome in murine macrophages [106].

Both NLRP3 and ASC have physical interactions with bruton tyrosine kinase (BTK), and NLRP3 inflammasome activation is inhibited by BTK blocking. BTK participates in TLR and B cell receptor signaling [107].

Activation of the NLRP3 inflammasome appears to occur in two steps [108]. This two-step process consists of priming and activation signals.

First, there is a priming or initiating signal in which a large number of PAMPs or DAMPs are recognized by TLRs. The recognition process initiates signaling, as mediated by nuclear factor-kappa B (NF-κB), which in turn boosts transcription of inflammasome-related components like proIL-18, proIL-1β, and dormant NLRP3 [109]. The different PAMPs and DAMPs are represented by extracytoplasmic ATP binding to P2X7R, bacteriotoxin, and a complement membrane attack complex (MAC). This binding results in the phagocytosis of nondegradable particulate matter (silica, alum, etc.) and the creation of cell membrane pores. Through direct transmembrane transport, this mechanism causes K^+^ efflux, which encourages the NLRP3 inflammasome to assemble and become activated [110].

**Figure 3:**
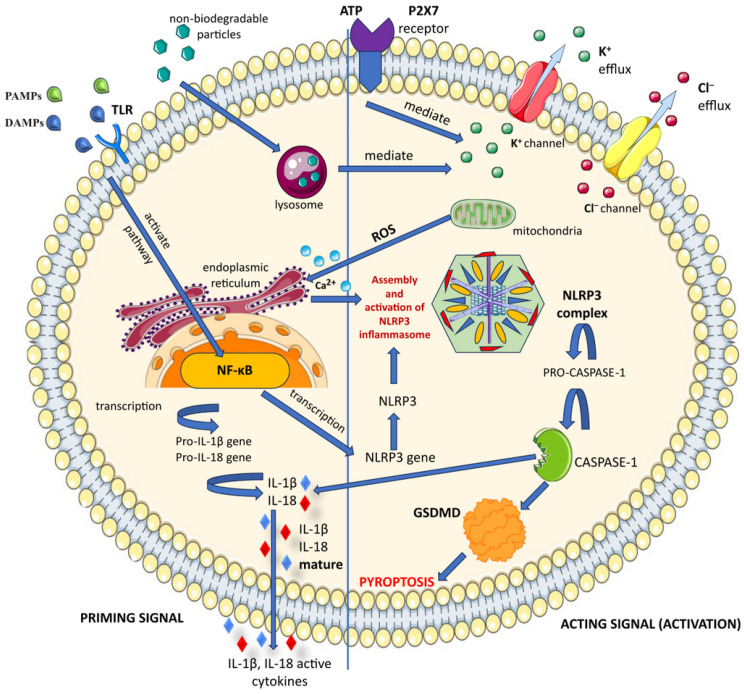
Canonical inflammasome activation. Legend: ATP, Adenosine triphosphate; DAMP, Damage-associated molecular pattern; GSDMD, Gasdermin D; NF-κB, Nuclear factor-kappa B; NLRP, Nucleotide-binding domain, leucine-rich-containing family, pyrin domain-containing; PAMP, Pathogen associated molecular pattern; P2X7R, P2X purinoceptor 7; ROS, Reactive oxygen species; TLR, Toll-like receptor.

When the NLRP3 inflammasome is activated, it triggers the breakdown of pro-caspase-1 into active caspase-1. This process converts pro-IL-1β and pro-IL-18 into mature forms of IL-1β and IL-18, respectively (Figure 3). Additionally, the endoplasmic reticulum (ER) responds to the production of ROS by mitochondria and releases Ca^2+^ [111].

Purified recombinant gasdermin D (GSDMD) was shown to be cleavable by caspase-1 in its active tetramer form, yielding a 22-kDa C-terminal fragment and a 38-kDa mature cleavage product. Through its N-terminal product, GSDMD primarily causes extensive cell death with apparent pyroptosis morphology and regulates IL-1β secretion without interfering with its maturation [112,113]. The activation of the NLRP3 inflammasome is thought to be dependent on changes in intracellular ion concentrations, including K^+^ outflow, Ca^2+^ influx, and Cl^−^ outflow [114].

The oligomerization of NLRP3 and subsequent complex formation of NLRP3, ASC, and procaspase-1 constitute the second stage of inflammasome activation. This initiates the maturation of IL-1β and IL-18, as well as the conversion of procaspase-1 to caspase-1 [115]. The innate immune system’s multiprotein platforms, or inflammasomes, depend on the ASC protein for their proper functioning. These inflammasomes become active in reaction to internal cell damage or infections when various inflammasome receptors start to act. When different inflammasome receptors initiate the formation of ASC specks, it promotes the activation and recruitment of procaspase-1. This triggers pyroptotic inflammatory cell death and the release of pro-inflammatory cytokines [116].

### 3.5. NLRP3 Non-Canonical Activation

Atypical NLRP3 inflammasome activation is the term for a phenomenon in which LPS not only acts as a trigger for inflammasomes, but also directly activates caspase-11, without the need for TLR4, to cause pyroptosis [117,118].

**Figure 4:**
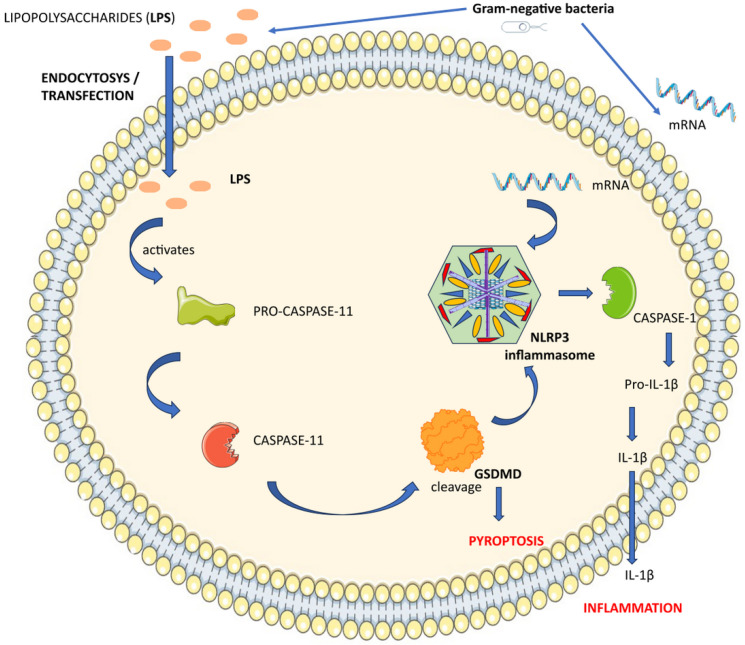
Non-canonical inflammasome activation. Legend: GSDMD, Gasdermin D; IL, Interleukin; LPS, Lipopolysaccharide; NLRP, Nucleotide-binding domain, leucine-rich-containing family, pyrin domain-containing; RNA, Ribonucleic acid.

The NLRP3 inflammasome primarily activates through a noncanonical pathway spurred by LPS present on the outer membrane of Gram-negative bacteria. Even in the absence of TLR4, LPS has the ability to penetrate the host cell’s cytoplasm directly through endocytosis or transfection [119]. Intracellular LPS binds to pro-caspase-11, causing it to become active and further trigger GSDMD cleavage. The NLRP3 inflammasome is activated and pyroptosis is caused by the amino-terminal GSDMD fragment [120]. For the noncanonical activation of the NLRP3 inflammasome in macrophages, LPS and mRNA from Gram-negative bacteria are required. Following LPS’s attachment to pro-caspase-11, NLRP3 locates the bacterial mRNA. The interaction between pro-caspase-11 and NLRP3 enhances the non-canonical activation of the NLRP3 inflammasome [121]. Activation of the NLRP3 inflammasome leads to the release of proinflammatory cytokines, such as IL-1β (Figure 4). According to a different study, the lysosomal membrane’s stability was altered by the caspase-11 activator cathepsin B after LPS-induced alterations [122].

GSDMD is required for the noncanonical NLRP3 inflammasome to activate. GSDMD-induced pyroptosis triggers the release of IL-1β, which intensifies local inflammation [123]. An enhanced signaling loop is produced by the mutual reinforcement of GSDMD and NLRP3 [120]. In the future, novel therapeutic compounds may target this loop.

These results demonstrate the essential function of the inflammasome, which interacts with various cellular and molecular pathways to manage attacks and threats to the host.

The inflammasome is a multiprotein complex crucial for immune responses. It consists of sensor molecules like NLRP3, NLRC4, NLRP1, and AIM2, adaptor proteins, and pro-caspase-1. Activation of the inflammasome leads to the processing of proinflammatory cytokines and pyroptotic cell death, aiding in host defense against infections.

## 4. Inflammasome and Innate Immune System

As the primary defense mechanism of the host during infections, the innate immune system plays a vital role in the early detection of invading pathogens and the initiation of a proinflammatory response [124]. Crucial to this response is the recognition of evolutionarily conserved pathogen structures, known as PAMPs, by a limited set of germline-encoded PRRs within the innate immune system. Among these PRRs, the TLR family has been extensively studied [125].

An innate immune response known as inflammation, which is mostly mediated by macrophages, is the body’s defense mechanism against pathogens such as bacteria, viruses, protozoa, and fungi. Swelling, redness, heat, loss of function, and pain are the hallmarks of inflammation [97]. Numerous inflammasomes have been found, including AIM2, NLRP1, NLRP3, NLRC4, and NLRP3 [126].

Inflammasome activation plays a crucial role and is orchestrated by the innate immune system, with recent advances in the understanding of the intricate macromolecular processes involved [101]. While the exact role of inflammasomes in autoimmunity remains somewhat unclear, it is evident that the innate immune system significantly influences autoimmune responses [127]. Additionally, a T cell-independent phase has been proposed as a critical element in the initiation of RA [128].

Recent studies have shed light on the potent induction of inflammatory responses instigated by inflammasomes located intracellularly, as well as protein complexes that spark inflammation within macrophages by triggering GSDMD-mediated pyroptosis and the secretion of pro-inflammatory cytokines, particularly IL-18 and IL-1β, in a caspase-1-dependent manner [90,129].

In cases of chronic inflammation, inflammatory responses persist over extended periods, lasting from weeks to years, characterized by repetitive cycles of tissue damage and healing, ultimately leading to significant tissue impairment. Chronic inflammation poses a notable risk for the development of inflammatory conditions or AIDs, encompassing a diverse range of conditions marked by persistent inflammation targeting self-tissues [130].

Inflammasomes are crucial components of the innate immune system, detecting danger signals and activating inflammation to combat pathogens and repair tissues in inflammatory diseases.

## 5. Inflammasome Involvement in Autoimmune Disorders

**Figure 5:**
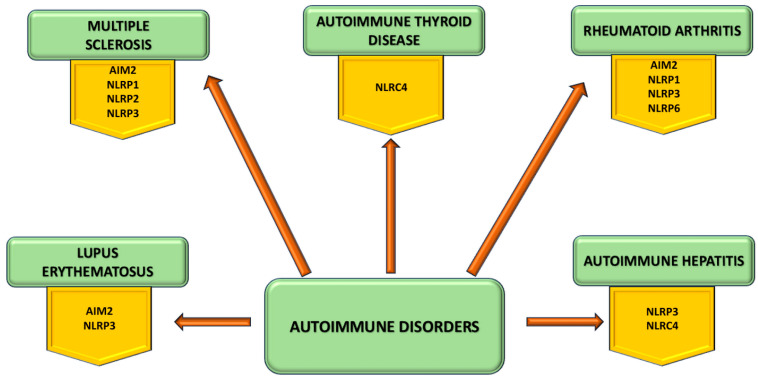
Inflammasomes involved in autoimmune diseases. Legend: AIM2, Absent in melanoma 2; NLRC4, NLR family CARD domain-containing protein 4; NLRP, Nucleotide-binding domain, leucine-rich-containing family, pyrin domain-containing.

Different types of inflammasomes are linked to numerous inflammatory disorders (Figure 5).

Out of these, NLRP3 stands out as the most extensively researched NLR. It is not likely for all these stimuli to be directly sensed, as NLRP3 can be triggered by a broad range of pathogens and their associated molecules. Rather, the prevailing consensus is that these signals all point to a common molecular event that specifically triggers NLRP3 [131]. The nucleotide-binding oligomerization domain-like receptor (NLR) family includes the NLRP3 protein. The pyrin domain (PYD) is located at the amino terminus of the NLRP3 protein, the nucleotide-binding domain (NACTH/NBD) is located in the center domain, and the LRR domain is located at the carboxyl terminus [87].

Nevertheless, when the NLRP3 inflammasome is excessively activated, it leads to heightened inflammation and unnecessary harm to the host, placing the body in a pathological condition [132]. The activation of the NLRP3 inflammasome not only accelerates the pathological progression of cardiovascular diseases but also worsens vascular endothelial dysfunction and oxidative stress [133].

Inflammasome NLRP3 is necessary for adaptive immunity. It produces large amounts of IL-1β and IL-18 when activated, which sets up a cytokine environment that causes naive T cells to differentiate into effector and memory T cells. This activation ultimately triggers the onset of adaptive immunity [134]. An overactivation of adaptive immunity determines autoimmune disorders.

The activation of the NLRP3 inflammasome is initiated by multiple upstream signals, such as K^+^ efflux, Cl^–^ efflux, Ca^2+^ flux, mitochondrial dysfunction, ROS production, and lysosomal damage [135]. Mitochondrial dysfunction occurs due to the release of mitochondrial DNA or cardiolipin, as well as the release of cathepsins into the cytosol following lysosomal destabilization [136].

When the NLRP3 inflammasome is activated, it leads to the release of IL-18 and IL-1β. The NLRP3/IL-1 pathway plays a crucial role in the body’s immune response and overall immune system function [132].

### 5.1. Inflammasome in Multiple Sclerosis

The NLRP3 inflammasome and MS occurrence are closely associated [137]. Recent research has shown that alterations in the genes of NLRP3-related molecules are associated with susceptibility to MS. One study examined the relationship between NLRP3 single nucleotide polymorphisms and MS susceptibility, finding that NLRP3 polymorphisms play a critical role in MS [138].

NLRP3 inflammasome-related molecules play a role in the pathophysiology of MS. Research indicates heightened levels of ASC, caspase-1, and IL-18 in the blood of MS patients, along with elevated expression of NLRP3 and IL-1b genes in MS plaques [139,140].

Furthermore, it has been observed that caspase-1 expression increases at the mRNA and protein levels. Furthermore, inhibiting caspase-1 has the potential to suppress inflammasome activation, thereby mitigating the severity of experimental autoimmune encephalomyelitis, an animal model frequently utilized to study MS [141].

Peripheral immune cell infiltration may result from compromised CNS barriers in the early stages of MS, and IL-1b facilitates CNS barrier degradation [137]. Tight junctions and adherens junctions, which are junctional components of CNS barriers, are dysregulated by immune mediators, and proinflammatory cytokine secretion serves as an additional catalyst for peripheral immune cell infiltration [142]. This process involves endothelial cells as well as CNS astrocytes, and activated astrocytes can release proinflammatory cytokines that harm endothelial cells’ tight junctions. Furthermore, MS develops because leukocytes are drawn into the CNS by chemokines released by activated astrocytes [143].

In the NLRP3 inflammasome activation in MS, the main cell types that are involved are astrocytes, microglia, and CD4+ T cells. The proinflammatory molecules activate infiltrated CD4+ T cells, thus amplifying the neuroinflammation process of microglia [144]. At the same time, microglia could secrete chemokines that recruit immune cells. By infiltrating the CD4+ T cells, the body triggers a cascade of proinflammatory cytokines that activate microglia. This activation leads to inflammation and can have serious consequences. It is important to understand the mechanisms behind this process to develop effective treatments and prevent long-term damage [145].

The neurotoxic A1 phenotype in MS is caused by the activation of astrocytes by the NLRP3 inflammasome in activated microglia, which exacerbates cognitive deficits [146].

The NLRP3 inflammasome, an essential part of innate immunity, facilitates the synthesis of IL-1b and IL-18, boosts peripheral immune cell infiltration into the central nervous system, and modifies T and B cell function. While the majority of research indicates that MS is primarily an immune disease involving T cells, there is increasing evidence that B cells play a critical role in the disease’s development [147]. T cell activation depends on B cells’ ability to deliver antigens to T cells. Despite a wealth of research on the subject, the relationship between B cells and the NLRP3 inflammasome in MS remains unclear, necessitating additional investigation to elucidate the underlying mechanism.

NLRP1 distinguishes itself as a unique inflammasome due to its structural features, possessing its own pyrin and CARD domain unlike NLRP3, AIM2, and pyrin. This unique structure allows NLRP1 to activate caspase-1 independently of ASC [148]. Variants of NLRP1 have been linked to MS pathogenesis, with a specific amino acid substitution (glycine to serine) associated with increased IL-18 and IL-1β production in familial MS cases [149].

NLRP2 inflammasome can be activated by IFN, ATP, and LPS, leading to inflammatory responses and influencing NLRP3 regulation. NLRP2 has diverse functions, including IFN inhibition and inflammation promotion outside of inflammasome activation, varying across cell types. Further biochemical research is required to fully comprehend the activation, assembly, and functional role of NLRP2 [150].

In addition to NLRP3, other inflammasome sensors like NLRP2 [151,152] have been observed in rodent and/or human microglia, astrocytes, neuronal cells, and pericytes, potentially impacting NDG progression [153]. NLRP2, while capable of inflammasome formation, may also exhibit anti-inflammatory effects. In 2004, Bruey et al. have suggested that NLRP2 functions as a modulator of NF-κB activation in macrophages, emphasizing its significance in regulating inflammatory signals across different cell types [150].

Although AIM2’s involvement in MS has been proposed, limited information exists regarding its specific role in this autoimmune condition [154]. AIM2 has been considered as a a potential therapeutic target in MS treatments, as it is downregulated in response to IFN-β therapy among MS patients [155]. The interplay between AIM2 and IFN-β may involve GSDMD, which can inhibit AIM2 and IFN-β responses to cytosolic DNA [156].

### 5.2. Inflammasome in Autoimmune Hepatitis

The exact mechanism linking NLRP3 inflammasome to the occurrence, development, and pathogenesis of autoimmune liver disease has yet to be fully understood.

AIH is a rare condition marked by high levels of transaminase, bilirubin, γ-globulin, and immunoglobulin G in the blood, along with the presence of anti-liver/kidney microsomal type 1 autoantibodies [157]. In order to stimulate macrophages and Kupffer cells to secrete IL-1β, IL-18, and TNF-α, Th cells generate and release IFN-γ and IL-2 during the progression of AIH [158,159].

Concanavalin A (ConA) is a substance that is commonly used to replicate AIH in rodents. This is because ConA causes activation of T lymphocytes and the production of proinflammatory cytokines. When ConA is administered to mice, it activates NLRP3 inflammasome, leading to the release of IL-1β and caspase-1. However, it is interesting to note that NLRP3-deficient mice were resistant to ConA-induced liver damage [159]. An alternative method for examining AIH in rodents is the use of trichloroethylene administration. This approach has been found to increase ROS production, leading to inflammasome activation and the development of the disease. These findings suggest that inflammasome activation is a significant factor in the pathology of AIH and plays a causative role in its progression [160,161].

NLRC4 is involved in driving human autoinflammatory diseases, characterized by systemic or organ-specific inflammation unrelated to infection, cancer, or specific autoimmunity [162]. While NLRC4 shares activation mechanisms with other inflammasomes, similar to NLRP1, it can recruit procaspase-1 to the complex through CARD–CARD interactions. The CARD domain of NLRC4 directly engages with pro-CASP1 to trigger CASP1 activation, with ASC aiding in this process positively [163]. Notably, the NLRC4 inflammasome is vital in bacterial infections within the liver, with NLRC4-mediated IL-1β release associated with liver inflammation [164].

### 5.3. Inflammasome in Autoimmune Thyroid Disease

AITDs refer to a group of thyroid diseases caused by autoimmune disorders that are characterized by damage to the thyroid tissue, with GD and HT being the primary types [104]. Guo et al. were the first to propose that multiple inflammasomes, including AIM2, NLRP1, NLRP3, NLRC4, play a crucial role in the development of AITDs [165]. An excessive amount of iodine can trigger pyroptosis activity in thyroid follicular cells through the ROS-NF-κB-NLRP3 pathway, which may contribute to the development of HT [166].

### 5.4. Inflammasome in Systemic Lupus Erhythematosus

An essential part of SLE development is played by the NLRP3 inflammasome. Studies have suggested that there may be a dysregulation of inflammasome activation in lupus, and they have also shown that IL-1β is produced in greater amounts in the kidneys of human monocytes and mice at risk of the disease, respectively, while IL1b gene expression is elevated [167,168]. New insights into the pathogenesis of SLE were revealed by the discovery of inflammasomes and their role in IL-1β secretion, even though the precise mechanism for these findings was unclear at the time [169]. Patients with SLE appear to have higher propensity for inflammasome activation in their monocytes and macrophages [170].

NLRP3 provides the most direct evidence of the various mechanisms by which inflammasomes are involved in the pathogenesis of SLE. Research has demonstrated that NLRP3 is implicated in the pathophysiology of SLE, mediates the pathogenicity of autoantibodies and type-I interferon (IFN-I), and interacts with NF-κB and other pathways to control T and B cell differentiation [171]. Nevertheless, it uses distinct signaling pathways to affect NLRP3 in its interaction with IFN-I, while NLRP3 prevents IFN-I signal transduction [172,173]. Thus, the function of NLRP3 in SLE patients exhibiting markedly aberrant IFN-I activation remains uncertain.

AIM2 is one of the p200 protein family members that recognizes intracellular double-stranded nucleic acids. When the AIM2 inflammasome detects intracellular nucleic acids, it assembles and activates, which causes caspase-1 to activate, pyroptosis to occur, and macrophages to release IL-1β [89,90]. The AIM2 protein is maybe the most well-known non-NLR capable of forming an inflammasome. However, it raises a crucial question about its ability to distinguish between bacterial or viral double-stranded DNA and self-DNA. This is because the inflammasome’s DNA-sensing ability has been linked to several disorders, such as psoriasis and SLE [174]. In a lupus mouse model, researchers found that suppression of macrophage activation and inflammatory responses led to a remarkable improvement in SLE symptoms. This was achieved through the inhibition of AIM2 expression, which played a crucial role in regulating the immune system response and reducing inflammation. The results highlight that targeting AIM2 could be a potential therapeutic strategy for managing SLE in humans [175].

### 5.5. Inflammasome in Rheumatoid Arthritis

RA is a serious lifelong AID and represents the inflammatory/AID with the highest prevalence worldwide; thus, the involvement of inflammasomes in the disorder has been actively studied [97]. Based on available data, activation of the inflammatory cascade is a risk factor for the RA pathogenesis.

Completed blood cells from patients with active RA have higher basal expressions of NLRP3, ASC, caspase-1, and pro-IL-1β compared to healthy individuals [176]. One of the essential pro-inflammatory cytokines linked to the pathophysiology of RA is IL-1β, which is secreted, and pyroptosis is induced by caspase-1, an effector molecule in the inflammasome [89,90].

In order to determine whether polymorphisms in the NLRP1 gene locus are associated with RA susceptibility, genetic research on the NLRP1 inflammasome linked to RA pathogenesis revealed that NLRP1 gene polymorphism is a risk factor for RA, causing upregulation of NLRP1 gene expression [177].

Genetic studies of RA inflammasomes have established the role of NLRP1 in RA pathogenesis. Zhang et al. carried out research to investigate how 11β-hydroxysteroid dehydrogenase 1 (11β-HSD1) affects the pathophysiology of RA. Their results showed that joint destruction and synovial inflammation associated with arthritis were significantly reduced when 11β-HSD1 activity was inhibited by its specific inhibitor, BVT-2733. This was accomplished by lowering the blood level of IL-1β and inhibiting the NLRP1 inflammasome’s assembly in collagen-induced arthritic mice compared to the control groups [104,178].

The NLRP3 inflammasome becomes hyperactive when normal mononuclear cells are cultured in RA serum, which increases the release of inflammatory cytokines like TNF-α, IL-1β, and IL-6. These alterations promote pyroptosis in mononuclear cells [179]. Further research was conducted on the NLRP3 inflammasome’s activation in RA patients. Thus, it was shown that RA patients produced NLRP3 inflammasome components and that patients with active RA had increased intracellular levels of NLRP3 inflammasome components, such as ASC, NLRP3, pro-IL-1β, active caspase-1, as well as increased secretion of IL-1β [176].

The NLRP3 inflammasome enhances the adaptive immune dysfunction of RA by promoting Th17 cell differentiation [180]. It has been established that Th17 is essential to the NLRP3 inflammasome’s downstream cascade reactions in RA. The Fas-associated death domain, a crucial adaptor molecule in inflammation and innate immunity, can also be secreted through the NLRP3 inflammasome [181].

NLRP6, characterized by molecular sensor structural motifs, is drawn to the cytosolic “specks” created by ASC oligomerization, thereby triggering procaspase-1 activation. Initially identified by Grenier et al. in 2002 through studies with human cell lines, the co-expression of NLRP6 (PYPAF5) and ASC was found to induce caspase-1 and NF-κB activation [182]. This led to the assumption that NLRP6 could potentially assemble an inflammasome like other NLR family members, although concrete in vivo evidence was lacking [183]. While much of the research exploring NLRP6 expression and function has been conducted in mouse models, studies involving human patients are limited. Notably, an anti-inflammatory function of NLRP6 has been observed in RA patients. Lin et al. conducted a study and discovered that NLRP6 was downregulated at both transcriptional and translational levels in synovial tissues and fibroblast-like synoviocytes (FLS) compared to osteoarthritis patients [184]. When NLRP6 was silenced in FLS from RA patients, there was an increase in proinflammatory cytokine production due to heightened NF-κB activity in response to TNF-α [183].

Concurrently, LRP3-associated AIM2 inflammasomes, serving as cytoplasmic receptors, have emerged as a focal point in recent research on RA pathogenesis. In RA joints, the formation of pannus is attributed to vascular hyperplasia, creating a hypoxic microenvironment [185]. Hypoxia within the joint leads to damage in mitochondrial or nuclear DNA. Given the proximity of mitochondrial DNA to the respiratory chain, it is more susceptible to oxidative stress-induced damage. RA patients exhibit elevated mtDNA levels in both plasma and synovial tissue compared to healthy controls, rendering them more prone to AIM2 inflammasome activation [186,187].

Inflammasomes, activated by NLR receptors, drive cytokine release in autoinflammatory disorders. The NLRP3 inflammasome plays a crucial role in autoimmune disorders by regulating inflammatory responses and promoting the production of inflammatory cytokines, contributing to pathophysiological signatures in different autoimmune disorders.

## 6. Inflammasome as a Therapeutic Target

The improper functioning of inflammasomes has been implicated in the development of neurodegenerative diseases and metabolic disorders.

Targeting the inhibition of the NLRP3 inflammasome could be a useful therapeutic strategy for autoimmune diseases, as this enzyme’s overactivation is a key factor in autoimmune responses. Previous studies have identified a variety of inhibitors of the NLRP3 inflammasome, including indirect inhibitors of ASC, caspase-1, the IL-1 signaling pathway, and pathways connected to the NLRP3 inflammasome, as well as direct inhibitors of the NLRP3 protein [132].

There is a lot of interest in inhibiting inflammasomes to treat various diseases. In particular, there is a focus on treating autoinflammatory syndromes that are caused by abnormal inflammasome activation due to genetic factors. Recent advancements in pharmaceuticals that inhibit cytokines and inflammasomes have helped in treating AIDs. As research in this area evolves, scientists are exploring methods to connect inflammasome activity with autoimmune responses. There are two main categories of therapies that can be employed for this purpose: inhibiting cytokines to block the result of inflammasome activity or inhibiting the inflammasome itself. Suppressing the inflammasome can prove advantageous in addressing both cytokine and non-cytokine-associated functions of the inflammasome that contribute to the progression of diseases.

Numerous agents have been developed to target inflammasome products IL-1β and IL-18 for treating AIDs. These include the IL-1 receptor antagonist anakinra, the IL-1β antibody canakinumab, and the IL-1 receptor decoy rilonacept among others, such as IL-18–binding protein, soluble IL-18 receptors and anti–IL-18 receptor monoclonal antibodies [188,189]. Additionally, glyburide, a common type 2 diabetes treatment, was the first compound identified as inhibiting NLRP3 [190].

Various small-molecule inhibitors that target NLRP3 along with NLRP1, NLRC4, or AIM2, such as parthenolide, auranofin, and isoliquiritigenin, as well as CRID3, Bay 11-7082, 3,4-methylenedioxy-β-nitrostyrene, cyclopentenone prostaglandin 15d-PGJ2 and 25-hydroxycholesterol (25-HC), have been studied, but further assessment is required for their in vivo efficacy. Most of these inhibitors are repurposed pharmacological agents designed to target the inflammasome [101].

Effective therapeutic strategies involve inhibiting NLRP3 oligomerization, with compounds like β-hydroxybutyrate (BHB), oridonin, tranilast, and CY-09 showing promise. Inhibiting active caspase-1 can be achieved with VX765 or similar drugs. Parthenolide can also inhibit the NF-κB signaling pathway, which prevents the priming of NLRP3 inflammasome. To inhibit the activation of the NLRP3 inflammasome, alternative approaches involve controlling ion flux using medications such as BHB and NSAIDs, blocking the IL-1 signaling pathway downstream with the IL-1 blockade, and promoting autophagy/mitophagy to eliminate triggering factors, constituents, and impaired mitochondria. Finally, M1 and other similar agonists have been found to help achieve this effect [132].

Furthermore, a range of compounds, including BHB, oridonin, tranilast, CY-09, parthenolide, and Bay 11-7082, directly target NLRP3 proteins [191].

Researchers are still investigating the specific way that BHB, a ketone body, disrupts NLRP3 inflammasome activity by inhibiting K^+^ efflux while decreasing ASC oligomerization and speck formation.

It also was suggested that IFN-I can hinder inflammasome activation through mechanisms not yet fully understood [172]. Recent studies have shed light on the impact of an IFN-stimulated gene product, cholesterol 25-hydroxylase (Ch25h), in suppressing IL-1b transcription and the activation of NLRP3, NLRC4, and AIM2 inflammasomes, implying a broad inhibitory influence on various inflammasomes. Additionally, the Ch25h substrate 25-HC has demonstrated the ability to impede NLRP3 inflammasome activation and the production of IL-1β [192].

In SLE, specific inflammasomes are associated with distinct organ damage, underscoring the need to select inflammasome inhibitors based on the disease’s clinical manifestations. The NLRP3 inflammasome is a potential target for therapy because of its involvement in diseases such as erythema, nephritis, and organ damage. It is crucial to tailor the selection of an inflammasome inhibitor based on the patient’s clinical profile, especially when taking organ involvement into account. For example, in patients with lupus nephritis (LN) accompanied by arthritis or erythema, an NLRP1 inflammasome inhibitor could be advantageous. Conversely, targeting the NLRP3 inflammasome might be more effective for LN patients with neuropsychiatric SLE [171].

Being a strong inhibitor of the NLRP3 inflammasome, MCC950 (diarylsulfonylurea compound) is the one that has been tested the most. Without influencing the NLRP1, AIM2, or NLRC4 inflammasomes, its specificity is noted because of the NLRP3 inflammasome target. MCC950 efficiently inhibits the activation of the NLRP3 inflammasome and lowers the secretion of IL-1β in human and mouse macrophages by blocking ASC oligomerization [193]. Nevertheless, concerns regarding liver toxicity have limited its application, as highlighted in a phase II clinical trial for RA [94].

IL-1β and IL-18, potent pro-inflammatory cytokines and primary outputs of the activated NLRP3 inflammasome, play crucial roles in the development of various inflammatory and AIDs. Consequently, inhibiting IL-1 presents a powerful treatment strategy that has been applied to numerous conditions related to NLRP3. While effective in managing NLRP3-driven inflammatory diseases and AIDs, the use of IL-1 blockade remains limited, partly due to frequent injections and the potential infection risks [132].

Nonsteroidal anti-inflammatory medications (NSAIDs) are often used during the acute stages of RA and spondyloarthritis to alleviate inflammation and joint discomfort. NSAIDs work by blocking Cl^–^ channels, which prevents the activation of the NLRP3 inflammasome [194].

Further clinical validation is necessary, but exploring the blockages of ROS, P2X7R, and K^+^ channels may provide additional helpful approaches. The compelling importance of NLRP3 as a target for the treatment of inflammatory and autoimmune diseases highlights the urgent need to develop highly specific and potent NLRP3 inflammasome inhibitors with as few adverse effects as possible [132].

Additionally, in investigating the blockage of K^+^ channels, P2X7R, and ROS could offer other useful strategies, although further clinical verification is required. The intriguing significance of NLRP3 as a target for treating autoimmune and inflammatory illnesses emphasizes the critical need to produce extremely effective and precise NLRP3 inflammasome inhibitors with minimum side effects.

Synthetic medications tend to have harsher effects than bioactive compounds, which include both physiologically essential molecules and active components obtained from plants [195]. Significant regulatory effects on NLRP3 may occur through the prevention of NLRP3 inflammasome activation or the manipulation of related signaling pathways, according to recent studies, even if the precise roles and target sites of many bioactive compounds are still unknown [196].

It is essential for modulating the biological effects of vitamin D and controlling immune responses linked to inflammatory and autoimmune illnesses that the vitamin D receptor (VDR), which is expressed in different immune cells, functions [197]. VDR plays an important role by directly binding to NLRP3 and preventing NLRP3 inflammasome activation by suppressing BRCC3-mediated ubiquitination [198]. Additionally, vitamin D exhibits promise in mitigating kidney damage in lupus mice models, by suppressing NF-κB and MAPK pathways while reducing autoantibody levels [199].

In autoimmune diseases, active substances such as flavonoids, 1,2,4-trimethoxybenzene, and uric acid can activate inflammasomes. In primary progressive MS patients, uric acid, which is present in higher concentrations in MS patients, activates the NLRP3 inflammasome pathway [200]. The anti-inflammatory properties of flavonoids target the activation of inflammatory RA in mice models, and these effects may be mitigated [169].

Some phytochemicals have demonstrated promise in blocking the NLRP3 inflammasome and managing LN, including curcumin, baicalein, sophocarpine, glycyrrhizic acid, and magnolol [201]. The epigallocatechin 3-gallate in green tea has been shown in mouse models to improve renal function by lowering ROS levels and inhibiting NLRP3 mRNA and protein [202].

Baicalein, a flavonoid compound derived from *Scutellaria baicalensis*, demonstrated an ability to inhibit the NLRP3 inflammasome, enhance Nrf2 activation, and downregulate the production of ROS in a murine model [201].

The natural quinolizidine alkaloid sophocarpine, derived from *Sophorae flavescentis*, has been shown to have a number of effects, including an anti-inflammatory one, and it lowers the levels of NLRP3 protein, caspase-1, and IL-1β in renal tissue [203].

Numerous effects, including immunoregulatory and antioxidative aspects, have been demonstrated by clinical and experimental studies involving glycyrrhizic acid derived from *Glycyrrhiza uralensis* [204]. This downregulates NF-κB signaling, reduces NLRP3 activation, and prevents severe renal injury [205].

Magnolol, a hydroxylated biphenyl compound derived from *Magnolia officinalis*, phosphorylates more IκB and NF-κB-p65 in mice, inhibiting NLRP3 activation, TNF-α secretion, and IL-1β secretion [206].

By blocking NLRP3 inflammasome activation, curcumin has been demonstrated to decrease renal inflammation, proteinuria, caspase-1 p20, and renal IL-1β in female mice [207].

These substances have the potential to be used as therapeutic targets for autoimmune diseases because of their critical roles in inflammasome activation.

In autoimmune disorders like SLE, RA, and other inflammatory diseases, inflammasomes play a crucial role. By modifying IL-1β production and immune cell behavior, targeting inflammasomes has shown promise in the treatment of autoimmune diseases. The variability of autoimmune diseases, such as SLE, can lead to a range of outcomes for inflammasome-targeting therapies. For autoimmune disorders to be effectively treated, it is essential to comprehend the distinct roles that various inflammasomes play in disease pathogenesis, their relationship to clinical phenotypes, and the possibility of therapeutic interventions.

## 7. New Biomarkers—Promising Strategies for the Management of Autoimmune Diseases

The preclinical management of autoinflammatory diseases can benefit from the use of novel biomarkers such as serum amyloid A, IL-18, and S100 proteins. These biomarkers can be used to monitor disease activity and predict patient outcomes [208]. Furthermore, the identification of cellular microparticles as a reservoir of bioactive compounds, alarmins, and self-antigens has yielded distinct perspectives on the state of the immune and vascular systems in autoimmunity, thereby presenting prospective avenues for therapeutic intervention [209].

Interleukin-6 and interleukin-18, which were discovered by cytokine profile analyses and inflammasome research, are novel biomarkers for the preclinical management of autoinflammatory diseases [210]. Progressions in the field of inflammasome biology have additionally resulted in the discovery of useful biomarkers, such as IFN-gamma and IL-18 levels, which facilitate the tracking and diagnosis of autoinflammatory diseases and pave the way for more focused interventions [211].

Other biomarkers for preclinical management of autoinflammatory diseases include protein arrays, immunoglobulin and T-cell-receptor-high-throughput sequencing, mass cytometry, and other markers of interest that help with diagnosis, prognosis, and treatment selection [212].

## 8. Conclusions

Molecular mimicry and inflammation are two key ideas in the study of molecular biology. As a natural reaction to infections, wounds, and other stimuli that jeopardize the body’s equilibrium, the immune system produces inflammation. On the other hand, molecular mimicry refers to the ability of certain microbial or environmental molecules to resemble self-antigens. This resemblance can lead to the production of autoantibodies, which can cause AIDs.

Because inflammation has the ability to undermine self-tolerance and trigger autoreactive immune cells, it is linked to molecular mimicry. Due to their molecular resemblance, during an infection the immune system may create antibodies directed against the invasive pathogen, which subsequently react with self-antigens. This reaction can lead to tissue damage and inflammation in the affected organs, leading to AIDs such as RA, MS, and lupus. Furthermore, inflammation can also increase the expression of self-antigens and the presentation of these antigens to T cells, further exacerbating the autoimmune response.

Therefore, it is essential to understand the mechanisms of inflammation and molecular mimicry to develop therapies that can modulate the immune system to prevent or manage AIDs. The NLRP3 inflammasome functions as a host defense mechanism that promotes pathogen clearance and equilibrium maintenance and can be activated by pathogenic invasions.

In conclusion, the NLRP3 inflammasome is a key player in the development and initiation of AIDSs, and it presents itself as a viable target for treatment interventions in these illnesses.

## 9. Future Directions

To develop effective treatments targeting inflammasomes in AIDs, it is crucial to conduct further research and gain a better understanding of their role in the body. It is important to find a balance between beneficial and harmful inflammasome activation, given its pivotal role in the body’s defense against microbial pathogens, as well as, potentially, in optimizing responses to vaccine adjuvants. Cytokines produced by the innate immune system influence the responses of the adaptive immune system. Therefore, not all inflammasome activations are deleterious, and any therapeutic interventions in this pathway must be meticulously assessed for their positive impact. As researchers gain greater insight into the mechanistic nature of inflammasomes, it is expected that new and more effective therapies will be developed for patients with inflammatory diseases.

## Data Availability

No new data were created or analyzed in this study. Data sharing is not applicable to this article.

## Abbreviations

5-HT	5-hydroxytryptamine
11β-HSD1	11β-hydroxysteroid dehydrogenase 1
AID	Autoimmune disease
AIH	Autoimmune hepatitis
AIM2	Absent in melanoma 2
AITD	Autoimmune thyroid disease
ALR	Absent in melanoma 2-like receptor
APC	Antigen-presenting cells
ASC	Apoptosis-associated speck-like protein containing a CARD
ATP	Adenosine triphosphate
BHB	β-Hydroxybutyrate
BTK	Bruton tyrosine kinase
CARD	Caspase activation and recruitment domain
Ch25h	Cholesterol 25-hydroxylase
CNS	Central nervous system
ConA	Concanavalin A
CTL	Cytotoxic T cell
DAMP	Damage-associated molecular pattern
DNA	Deoxyribonucleic acid
EBV	Epstein–Barr virus
ER	Endoplasmic reticulum
FasL	Fas ligand
FLS	Fibroblast-like synoviocytes
GD	Graves’ disease
GSDMD	Gasdermin D
HCV	Hepatitis C virus
HT	Hashimoto’s thyroiditis
IL	Interleukin
IFN-γ	Interferon-gamma
IFN-I	Type-I interferon
LN	Lupus nephritis
LPS	Lipopolysaccharide
LRR	Leucine-rich repeat
MAC	Membrane attack complex
MHC	Major histocompatibility complex
MS	Multiple sclerosis
Mt	Mitochondrial
NACTH/NBD	Nucleotide-binding domain
NEK7	NIMA-related protein kinase 7
NF-κB	Nuclear factor-kappa B
NIMA	Never in mitosis gene A
NOD	Nucleotide-binding and oligomerization domain
NLR	NOD-like receptor
NLRC4	NLR family CARD domain-containing protein 4
NLRP	Nucleotide-binding domain, leucine-rich-containing family, pyrin domain-containing
NSAID	Nonsteroidal anti-inflammatory drug
P2X7R	P2X purinoceptor 7
PAMP	Pathogen associated molecular pattern
PRR	Pattern recognition receptor
PYD	Pyrin domain
RA	Rheumatoid arthritis
RIG-I	Retinoic acid-inducible gene I
RLR	RIG-I-like receptor
RNA	Ribonucleic acid
ROS	Reactive oxygen species
SLE	Systemic lupus erythematosus
TCR	T cell receptor
Th	T helper
TLR	Toll-like receptor
TNF-α	Tumor necrosis factor-alpha
UVB	Ultraviolet radiation type B
VDR	Vitamin D receptor
